# Activation Efficiency and Restoration Effects of SBS Network-Repairing Regenerators on Aged Asphalt

**DOI:** 10.3390/ma19050888

**Published:** 2026-02-27

**Authors:** Mengmeng Jiang, Xin Yu, Ning Li, Jiandong Huang, Zhinan Cheng

**Affiliations:** 1 College of Civil and Transportation Engineering, Hohai University, Nanjing 210098, China; bella_0725@163.com (M.J.); lining24@hhu.edu.cn (N.L.);; 2 School of Traffic and Transportation Engineering, Changsha University of Science and Technology, Changsha 410114, China; 3 School of Civil Engineering, Guangzhou University, Guangzhou 510006, China

**Keywords:** aged SBS-modified asphalt, regeneration mechanisms, active regenerants, rheological properties, chemical structure, penetration performance

## Abstract

**Highlights:**

**What are the main findings?**

- Regenerants containing epoxy groups can effectively restore the fractured polymer chains of SBS following thermal-oxidative aging.
- The high and low-temperature performance of the recycled asphalt has been improved following the restoration of the fractured SBS polymer chains during aging.
- This regenerant is capable of restoring the viscoelastic properties of aged asphalt.
- Compared with commercially available regenerants, this regenerant exhibits a superior penetration effect.

**What are the implications of the main findings?**

- The recoverable aging of the SBS network structure indicates that the reclaimed asphalt pavement (RAP) from SBS-modified asphalt pavements can be reused in the original pavement or in high-performance layers requiring SBS-modified asphalt, without necessitating downgraded application.
- Improved permeability enables a greater quantity of regenerant to penetrate into aged SBS-modified asphalt during actual construction, thereby facilitating more extensive restoration of the degraded SBS modifier and significantly enhancing the performance of the recycled asphalt.

**Abstract:**

Although extensive research has been conducted on the regenerants for unmodified and SBS-modified asphalt, in-depth studies on the activation of regenerants to restore the SBS cross-linked network while preserving their diffusion performance have not yet been reported. This study quantitatively evaluated the activation effect of self-healing regenerants on SBS cross-linked networks by testing the activation degree of 6%, 8%, and 10% cross-linked networks with self-healing regenerants; the phase structure of SBS-modified asphalt before and after regeneration was examined using fluorescence microscopy (FM); the underlying mechanism of the reactive regenerant was elucidated by Fourier Transform Infrared Spectroscopy (FTIR) and Gel Permeation Chromatography (GPC); furthermore, the rheological response characteristics of the reactive regenerant and conventional regenerant were comparatively analyzed. The findings indicated that the SBS cross-linked network self-healing regenerant exhibited a more pronounced activation effect on aged asphalt. Specifically, when the dosage of the regenerant reaches 8%, its repairing effect on the cross-linked network becomes particularly significant. Reconstructing the cross-linked network structure of SBS-modified asphalt enabled the recovery of the viscoelastic properties of the recycled asphalt. Nevertheless, an excessive dosage of the regenerant failed to further enhance the cross-linked structure in a meaningful way and might even exert an adverse impact on the high-temperature performance of the recycled asphalt.

## 1. Introduction

In China, the total mileage of highways has exceeded 190,700 km. With the increase in pavement diseases on early constructed roads, maintenance projects are extensive, leading to the generation of approximately 230 million tons of RAP each year [1,2]. To comply with the policy directives of ‘green’ and ‘low-carbon’, and decrease the environmental impact of RAP processing and energy consumption, efforts are made to maximize the utilization of RAP while ensuring the enhanced durability of recycled asphalt mixtures [3,4,5].

The asphalt content in RAP material is generally in the range of 3% to 5%. Due to the effects of ultraviolet radiation, water, and thermal oxidation during service, the asphalt undergoes aging, characterized by a reduction in light components (aromatic and saturate) and an increase in asphaltene. This aging process can lead to the deterioration of asphalt performance. The restoration of the performance of aged asphalt is crucial for ensuring the road performance of recycled asphalt mixtures. The addition of regenerants is an effective method to restore the properties of aged asphalt binders. Currently, regenerants often employ component blending methods to restore the chemical composition, physical, and rheological properties of aged asphalt [6,7]. For example, waste bio-oil can effectively restore the colloidal structure stability and improve the rheological performance of aged asphalt binder. However, its application may lead to a significant decline in the high-temperature performance of the recycled asphalt binder [8,9,10]. The utilization of waste engine oil regenerants results in aged asphalt binder having improved elasticity and toughness [11]. In general, commonly used regenerants currently consist predominantly of high-aromatic oils, whose mechanism of action involves restoring the performance of aged asphalt by balancing chemical components. SBS-modified asphalt is commonly used in the surface layer of high-grade highways at present. In comparison to regular pavement petroleum asphalt, the aging of SBS-modified asphalt under the coupling effects of multiple factors involves both asphalt aging and the degradation of the SBS modifier. These methods mentioned above can only adjust the four components of aged SBS-modified asphalt, but are unable to repair the SBS modifier. Therefore, they are not suitable for aged SBS-modified asphalt, which is far from sufficient for the maintenance of high-grade road surfaces [12,13,14,15,16]. Bai et al. achieved performance restoration of aged SBS-modified asphalt by employing a low-viscosity regenerant. The results indicate that this regenerant significantly improves the low-temperature performance of the recycled SBS-modified asphalt, but there is a sharp decline in high-temperature performance [17]. Wang et al. evaluated the restorative effects of a regenerant rich in saturated and aromatic oils on the performance of recycled SBS-modified asphalt. Experimental results indicate that this regenerant enhances the cracking resistance of the recycled SBS-modified asphalt, but it does not have any impact on its microscopic phase structure. This is because the aging process of SBS-modified asphalt involves two components: the aging of the base asphalt and the degradation of the SBS modifier [18]. Common regenerants in the market can only restore the colloidal structure of the aged base asphalt through component adjustments, and they are unable to restore the microscopic phase structure of aged SBS-modified asphalt [19,20,21]. To sum up, the regeneration of aged SBS-modified asphalt involves not only the restoration of the performance of the aged base asphalt but, more crucially, the restoration of the microscopic phase structure of SBS.

**Figure 1:**
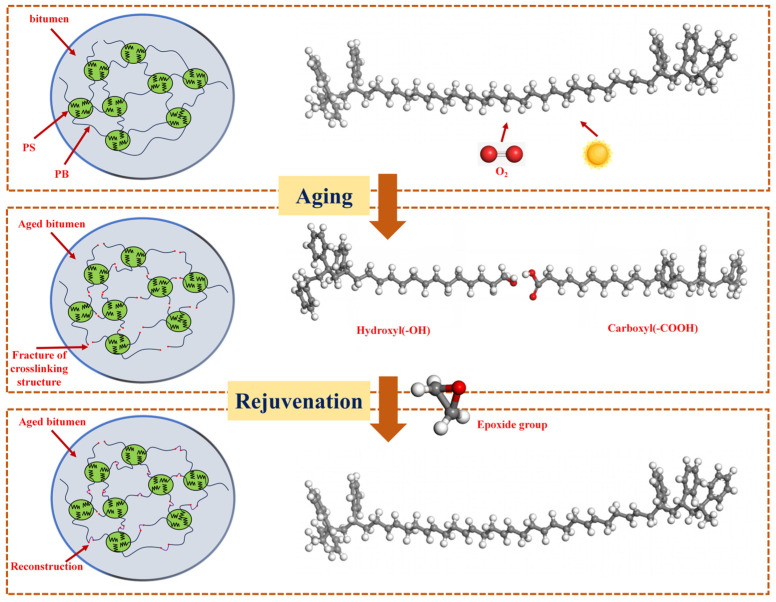
Structural damage and reconstruction diagram of SBS modifier.

Research indicates that the polybutadiene (PB) soft segments of SBS contain unsaturated double bonds, making them susceptible to oxidative cleavage under thermal oxidation, leading to severe damage to the cross-linking structure. After degradation of the SBS modifier, active cleavage fragments are produced, including terminal groups such as carboxyl and hydroxyl groups [22,23]. The reactive regenerant with active groups (such as epoxy groups) can react with the terminal groups of SBS segments, linking and reconstructing the broken PB segments. This process rebuilds the spatial network structure of the aged SBS modifier in asphalt [24]. The schematic diagram of the destruction and reconstruction of the SBS modifier structure is illustrated in Figure 1. Currently, research primarily focuses on supplementing the SBS modifier and employing various reactive materials to restore the degraded structure of SBS [25]. Among these methods, supplementing the SBS modifier tends to increase the overall viscosity of the regenerant, which is unfavorable for the ambient temperature spraying of the regenerant and its penetration onto the surface of RAP. On the other hand, reactive regenerants synthesized from various materials often face production challenges due to the demanding reaction conditions [26].

The paper chooses active epoxy-based material, penetrant, and highly penetrative waste engine oil as the base oil to prepare a high-activity reactive regenerant. On one hand, the active epoxy-based material can react with carboxyl and hydroxyl groups produced after the degradation of SBS modifier, which serves to restore the cross-linking structure in aged SBS-modified asphalt. On the other hand, the intermolecular forces in aged SBS-modified asphalt are strong. To enable the reactive regenerant to penetrate effectively and undergo a reaction, waste engine oil, supplemented with penetrant, enhances the penetration effect of the regenerant and strengthens the reaction between the regenerant and the active segments of the degraded SBS modifier. This paper investigates the impact of the regenerant prepared by waste engine oil, penetrant, and epoxy-based material on the microscopic phase structure and rheological response of aged SBS-modified asphalt. Furthermore, this paper evaluates the penetration effectiveness of the reactive regenerant. This study enhances the penetration capability of the cross-linking self-healing regenerant into the SBS polymer network, enabling deep infiltration into aged asphalt within reclaimed asphalt pavement (RAP) and facilitating covalent reconnection with a greater number of scission sites in the degraded SBS chains, thereby significantly improving the functional recovery of the SBS modifier. These findings contribute to enhancing the performance of aged SBS-modified asphalt on the surface of RAP and promote the high-quality utilization of RAP in maintenance projects.

## 2. Materials and Methods

### 2.1. Materials

#### 2.1.1. Preparation of the Aged SBS-Modified Asphalt Samples

**Figure 2:**
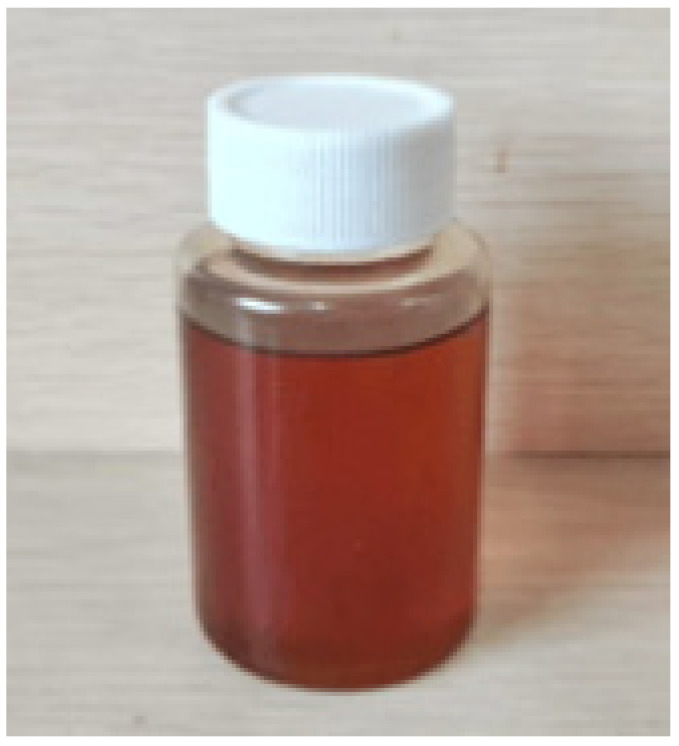
Schematic diagram of ZS-1 product.

SBS-modified asphalt is supplied by Nantong Tongsha Asphalt Technology Co., Ltd., Nantong, Jiangsu Province, China. The regenerant samples are shown in Figure 2. The base asphalt inside is South Korean S-Oil 70# base asphalt, and the performance indicators of the base asphalt are as shown in Table 1. The performance indicators of SBS-modified asphalt are shown in Table 2.

This paper employed laboratory-simulated aging of asphalt. SBS-modified asphalt was subjected to short-term aging (RTFOT) and long-term aging (PAV) according to ASTM Standard D1754, 2021 [27], and ASTM Standard D6521, 2022 [28], respectively. The results yielded laboratory-simulated aged SBS-modified asphalt, labeled as ASBS, and its physical performance indicators are presented in Table 3.

#### 2.1.2. Regenerants

To restore the spatial network structure of aged SBS-modified asphalt, the regenerant, labeled as ZS-1, as shown in the figure, was developed. ZS-1 employed waste engine oil as the base oil to rebalance the colloid structure of the aged asphalt binder. Among these components, the aromatic-to-saturated hydrocarbon ratio is approximately 3:1. To restore the structure of aged SBS, epoxidized soybean oil was added to the waste engine oil, and mechanical stirring was carried out at a temperature of 120 °C for 15 min with a rotational speed of 500 r/min. To ensure the effective restoration of the aged SBS structure by the active components, JFC was employed to enhance the penetration effectiveness of the regenerant. When the obtained sample was cooled to 70 °C, the penetrant was added, and then mechanical stirring was carried out with a rotational speed of 500 r/min for 15 min. At the conclusion of this process, the reactive regenerant was obtained. A comparison was made between ZS-1 and a commercially available regenerant, labeled as ZS-2. The ZS-2 instrument was acquired from Jiangsu Modern Road and Bridge Co., Ltd., Nanjing City, Jiangsu Province, China. The physical performance indicators of the two regenerants are presented in Table 4.

#### 2.1.3. Preparation of the Recycled SBS-Modified Asphalt Samples

**Figure 3:**
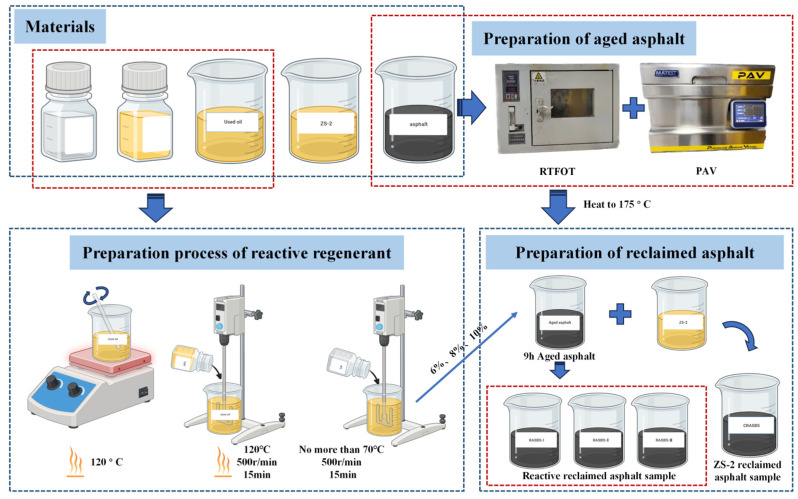
Sample preparation process of the regenerant and recycled asphalt.

When the ASBS sample was heated to 175 °C, ZS-1 was added at concentrations of 6%, 8%, and 10%, while ZS-2 was added at a concentration of 8%. The sample temperature was maintained at 175 °C, and mechanical stirring with a rotational speed of 500 r/min was conducted for 30 min to obtain four types of recycled SBS-modified asphalt samples. The samples prepared with ZS-1 were denoted as RASBS-I, RASBS-II, and RASBS-III, while the sample prepared with ZS-2 was denoted as RASBS-C. The sample preparation process is illustrated in Figure 3.

### 2.2. Measurement and Characterization

#### 2.2.1. Basic Physical Properties Test

Fundamental physical characteristics of the asphalt binders were evaluated strictly following ASTM protocols. Specifically, the softening point, penetration, ductility, and rotational viscosity were measured in accordance with ASTM D36 [29], ASTM D5 [30], ASTM D113 [31], and ASTM D4402 [32], respectively. To guarantee data reliability and minimize random errors, a triplicate testing strategy was implemented for each test group, and the arithmetic mean was reported as the final result.

#### 2.2.2. Temperature Sweep Test

The failure temperatures of asphalt samples were characterized using a Dynamic Shear Rheometer (DSR, TA Instruments, New Castle, DE, USA). Following the test methods outlined in ASTM D7175-23 [33]. Approximately 0.1 g of the sample was placed on the lower plate of the parallel plates, which were mounted on the rheometer. Afterwards, the starting temperature was set, and once the sample softened, the upper plate extruded a portion of the sample. After adjusting the sample, the gap between the parallel plates (25 mm) was set to 1mm. The temperature scan was conducted in the range of 52 °C to 94 °C, with a heating rate of 2 °C/min and a frequency of 10 rad/s. Steady-state shear viscous flow tests were performed on the samples in the rheometer’s steady-state shear mode. To ensure data accuracy, the test was repeated three times, and the arithmetic mean was adopted as the final result.

#### 2.2.3. Frequency Sweep Test

Frequency sweep allows for obtaining the linear viscoelastic parameters of asphalt at intermediate temperatures, which are highly sensitive to changes in asphalt composition and internal structure. Following the test methods outlined in ASTM D7175-23 [33]. The frequency sweep ranged from 0.1 to 100 Hz, and experiments were conducted at temperatures of 6 °C, 20 °C, 35 °C, 50 °C, 65 °C, and 80 °C. Two different specimen sizes were used to cover the entire temperature range: for temperatures below 35 °C, the parallel plates had a diameter of 8mm with a gap of 2 mm, while for temperatures above 35 °C, the parallel plates had a diameter of 25 mm with a gap of 1mm.

#### 2.2.4. MSCR Test

To characterize the pavement deformation behavior under simulated traffic loading, the MSCR test was performed on RTFOT-aged residues of all six asphalt variants using a Dynamic Shear Rheometer (DSR). Strictly adhering to the protocols defined in AASHTO T 350-14 and ASTM D7405-15 [34], the testing procedure involved two distinct stress levels (0.1 kPa and 3.2 kPa) at temperatures of 60 °C and 70 °C. For each stress level, the protocol consisted of 10 consecutive cycles, with each cycle comprising a 1 s creep loading phase followed by a 9 s recovery period. Key performance indicators, namely the percent recovery (*R*) and non-recoverable creep compliance (*J_nr_*), were derived using Equations (1) and (2). To guarantee experimental reliability, three independent replicates were tested for each condition, and the arithmetic mean was reported.

(1) $$J_{nr}(\sigma ,N)=\frac{\varepsilon_{r}-\varepsilon_{0}}{\sigma }$$

(2) $$R(\sigma ,N)=\frac{\varepsilon_{c}-\varepsilon_{r}}{\varepsilon_{c}-\varepsilon_{0}}\times 100$$

where *σ* denotes the applied stress level, set at 0.1 kPa or 3.2 kPa; *ε*_0_ denotes the initial strain at the beginning of each cycle, %; *ε_c_* denotes the strain at the end of the creep phase, %; *ε_r_* denotes the strain at the end of the recovery phase, %.

#### 2.2.5. BBR Test

To assess low-temperature creep behavior, BBR testing was performed across a temperature gradient ranging from −12 °C down to −27 °C (at 3 °C intervals). Deflection data were recorded at discrete loading durations of 8, 15, 30, 60, 120, and 240 s, respectively, to compute the creep stiffness (*S*) and relaxation rate (*m*-value). Following the test methods outlined in ASTM D6648-01 [35]. To ensure data accuracy, the test was repeated three times, and the arithmetic mean was adopted as the final result.

#### 2.2.6. FTIR Test

The changes in the chemical structure of the asphalt before and after the addition of the reactive regenerant were analyzed using an FTIR spectrometer (Nicolet IS20, ThermoFisher, Waltham, MA, USA) equipped with an Attenuated Total Reflectance (ATR) accessory with a diamond crystal (TensorII, Bruker, Karlsruhe, Germany). To confirm whether the epoxy-based material reacts with the regenerant or asphalt, the paper also conducted infrared spectroscopy on mixtures of asphalt, regenerant, and epoxy-based material. The testing wavelength ranged from 4000 cm^−1^ to 400 cm^−1^, with a resolution of 4 cm^−1^ and 32 scans.

#### 2.2.7. GPC Test

GPC can effectively assess the changes in the molecular weight distribution of recycled binder materials. Weigh the binder sample and dissolve it in 15 mL of tetrahydrofuran with a concentration of 2–3%. Then let the solution stand for 3 days to ensure complete dissolution of asphalt and SBS modifier in tetrahydrofuran. To remove impurity particles, aspirate 2 mL of the solution through a 0.45 mm filter into the sample bottle. Then, aspirate 0.5 mL of the processed solution into the GPC instrument. The experimental temperature is 25 °C, and the flow rate is 1.0 mL/min.

#### 2.2.8. Fluorescence Microscope Test

Melt the recycled asphalt to a liquid state at 175 °C and drop it onto a glass slide. Then, place it in an oven at 150 °C, keeping it level and stationary for 30 min to allow the asphalt to disperse evenly. Afterward, remove it and let it cool to room temperature. The distribution of SBS was observed using a fluorescence microscope (FM, DXY-1, Shanghai, China). Professional image processing software, such as Image-Pro Plus 6.0, and analysis techniques were employed for quantitative characterization of the scale and distribution of polymer phases in asphalt.

#### 2.2.9. Permeability Evaluation Test

Contact angle measurements were employed to simulate the penetration behavior of the regenerant into the aged asphalt binder on the RAP surface, thereby enabling a quantitative assessment of regenerant penetration efficacy. Aged asphalt was heated to 175 °C, after which 0.5 mL of the molten asphalt was transferred using a glass syringe into the sample tube of the contact angle tester. The sample tube containing asphalt was placed in an oven and heated at 140 °C for 30 min, after which 8% (by mass relative to aged asphalt) of the regenerant was added. The asphalt mixed with the regenerant, along with the test tube, was transferred to the testing equipment and maintained at 140 °C for 1 h to facilitate permeation.

**Figure 4:**
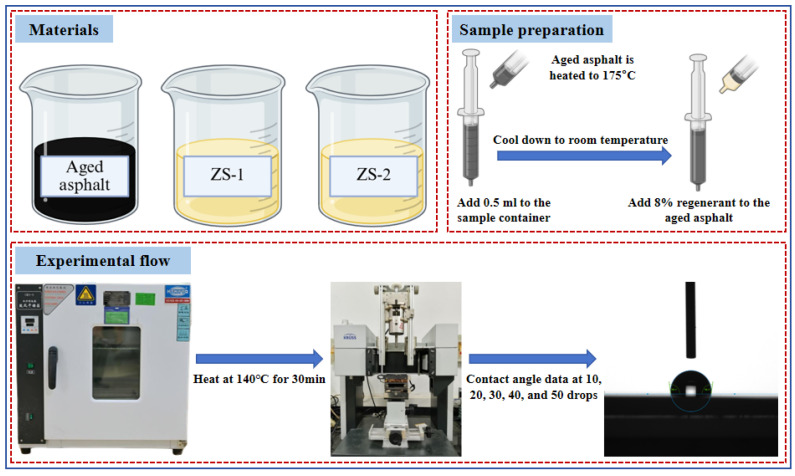
Evaluation method for regenerant permeability.

The contact angle measurement was performed using the DSA-100 optical contact angle analyzer manufactured by Krüss GmbH (Hamburg, Germany), which is equipped with a room-temperature module and a high-temperature module, as shown in Figure 4. The instrument consists primarily of a camera, illumination system, and dedicated analysis software, with a contact angle measurement accuracy of ±0.01°. The procedure of the contact angle test is as follows: Asphalt and glass slides were simultaneously placed at fixed positions in the temperature-controlled chamber of the contact angle analyzer for thermal equilibration. The instrument was adjusted to allow a molten asphalt droplet to slowly deposit onto the surface of the glass slide. To eliminate air bubbles present in the initial droplets, the first two droplets were discarded, and data recording began from the third droplet, with a total of 50 droplets tested. Contact angle measurements from the 10th, 20th, 30th, 40th, and 50th droplets were selected to evaluate the permeability of the regenerant and were designated as Layer 1, Layer 2, Layer 3, Layer 4, and Layer 5, respectively. The contact angle between the asphalt and the glass slide was automatically calculated by the instrument. The test temperature was maintained at 140 °C, with a total measurement duration of 1 min, during which contact angles were recorded at 1 s intervals, yielding 60 data points per measurement.

## 3. Results and Discussion

### 3.1. Study on Regeneration Mechanism of Reactive Regenerant

#### 3.1.1. Fluorescence Images

**Figure 5:**
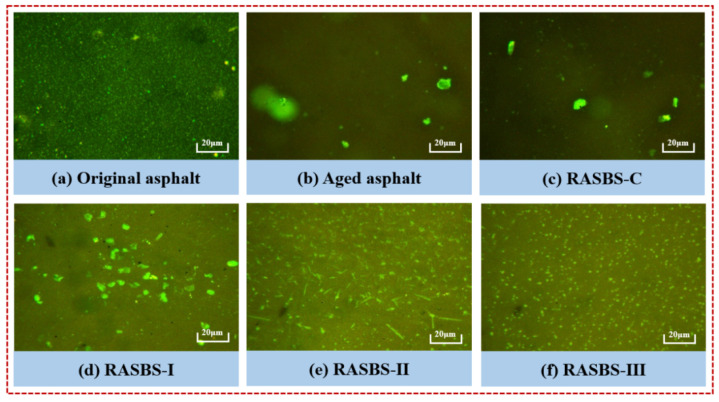
Phase structure diagram of asphalt samples.

The phase structure of SBS-modified asphalt was observed using fluorescence microscopy, including the original, aged, and recycled asphalt samples with different regenerant additions, as shown in Figure 5. In the original SBS-modified asphalt, the SBS phase is evenly distributed within the asphalt. After aging, the SBS phase in the SBS-modified asphalt is severely damaged, and the modifier aggregates significantly, leading to an increase in particle size. After adding 8% ZS-2, the morphological structure of SBS-modified asphalt did not make a difference, indicating that it cannot restore the degraded SBS modifier. Consequently, its ability to recover the performance of the recycled asphalt is limited.

To restore the phase structure of SBS-modified asphalt, the reactive regenerant is used to reconstruct the degraded SBS. As seen in Figure 5c–e, with the increase in the dosage of ZS-1, the spatial network structure of the recycled asphalt samples gradually recovers. In comparison with RASBS-I, RASBS-II shows a more significant restoration of the morphological structure. However, RASBS-II shows no significant difference compared to RASBS-III. The results indicate that reactive regenerant can effectively crosslink the degraded SBS modifier. Based on the theory of polymer solution and solubility parameter, the light oil components in the regenerant can effectively disperse the aggregated SBS modifier, further enhancing the spatial structure reconstruction of the degraded SBS modifier [36]. The results indicate that reactive regenerant can effectively crosslink the degraded SBS modifier. Based on the theory of polymer solution and solubility parameter, the light oil components in the regenerant can effectively disperse the aggregated SBS modifier, further enhancing the spatial structure reconstruction of the degraded SBS modifier.

#### 3.1.2. FTIR

**Figure 6:**
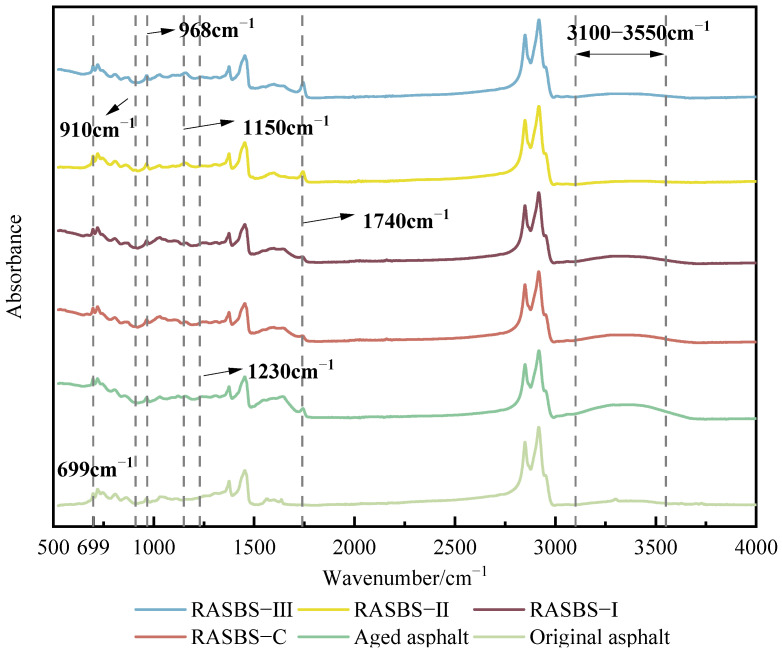
FTIR spectra of asphalt samples.

The impact of the reactive regenerant on the functional groups of aged SBS-modified asphalt was identified by FTIR. The infrared spectra of each sample are illustrated in Figure 6.

**Figure 7:**
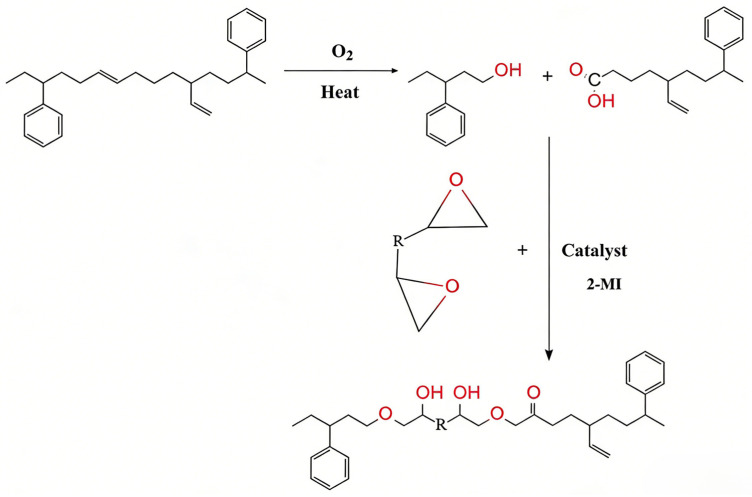
Reaction mechanism diagram of the reactive regenerant.

As illustrated in Figure 6 the characteristic absorption bands at 699 cm^−1^ and 968 cm^−1^ are assigned to the polystyrene (PS) and polybutadiene (PB) blocks of the SBS copolymer, respectively. The distinct attenuation of the 968 cm^−1^ peak following thermo-oxidative aging is attributed to the oxidation of unsaturated double bonds, confirming severe degradation within the PB phase. Meanwhile, the broad band spanning 3100–3550 cm^−1^, associated with hydroxyl stretching vibrations, intensifies significantly, suggesting an accumulation of reactive hydroxyl species during aging. In contrast to the reactive regenerant, the addition of ZS-2 induces no observable spectral shifts, implying it functions solely through physical component regulation without chemical interaction. Conversely, with the introduction of the reactive regenerant, the hydroxyl band diminishes progressively as dosage increases, evidencing the consumption of active hydroxyls via reaction with epoxy groups. Furthermore, the emergence of new peaks at 1102 cm^−1^ and 1740 cm^−1^ signifies the formation of ester linkages (ZS-1 reacting with carboxyls), while the 1250 cm^−1^ peak corresponds to ether bond (C–O–C) vibrations derived from the reaction with hydroxyls (refer to Figure 7. The mechanism involves epoxy ring-opening reactions with carboxyl and hydroxyl groups, generating ester and ether bonds, respectively. Notably, the secondary hydroxyls produced can facilitate further crosslinking with the degraded SBS, thereby reconstructing the spatial network and recovering binder performance. The absence of the characteristic epoxy peak at 910 cm^−1^ suggests complete conversion of the functional groups at the tested dosage. This analysis provides a qualitative verification of the reaction mechanism.

#### 3.1.3. GPC Analysis

**Figure 8:**
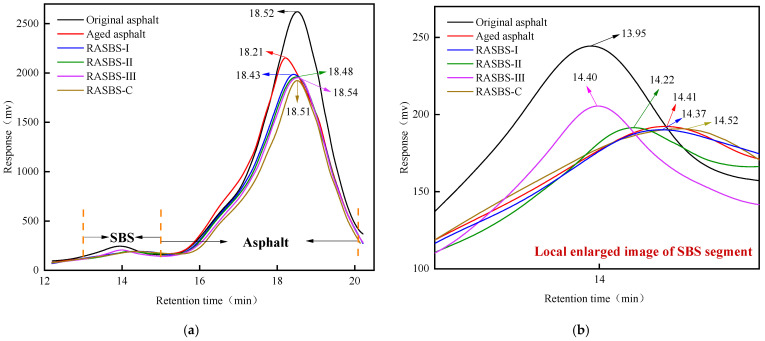
Molecular weight distribution schematic diagram for asphalt samples: (**a**) Molecular weight distribution of asphalt samples; (**b**) Local magnification of SBS segment.

Figure 8a illustrates the GPC elution profiles of the asphalt binders. A dominant peak is observable within the 15–20 min elution window, serving as a fingerprint for the binder’s molecular weight distribution (MWD). Relative to the virgin SBS-modified asphalt, the aged sample displays a peak migration toward an earlier time (18.21 min), signaling an increase in overall molecular size. This shift is mechanistically attributed to thermo-oxidative polymerization, which promotes the condensation of smaller molecules into larger aggregates. Conversely, the introduction of 8% ZS-1 and ZS-2 reverses this trend, displacing the peak to later elution times of 18.48 min and 18.51 min, respectively. This trajectory suggests a successful restoration of the aged binder’s MWD, primarily driven by the low-molecular-weight fractions in the waste engine oil that replenish the maltenes and re-stabilize the colloidal hierarchy. Notably, higher dosages of ZS-1 intensify this rightward shift. However, governed by the theory of colloidal stability, an equilibrium exists; thus, the supplementation of light components must be optimized, typically validated through macro-physical performance metrics.

As depicted in Figure 8a, a minor signal is detectable within the 13–15 min elution interval, which is identified as the high-molecular-weight SBS polymer. The magnified view in Figure 8b further elucidates the molecular weight distribution (MWD) characteristics of the modifier. Specifically, the characteristic peak for the virgin SBS-modified binder is located at 13.95 min, whereas the aged sample exhibits a rightward migration to 14.41 min. This delay in elution time signifies a reduction in molecular size, primarily driven by the oxidative scission of unsaturated double bonds within the polybutadiene (PB) blocks. Furthermore, the oxidation of allylic hydrogen atoms accelerates this thermal degradation process. Comparative analysis reveals that the ZS-2 regenerant induces negligible changes to the peak position. In contrast, the introduction of ZS-1 results in a distinct leftward shift to 14.22 min, with higher dosages correlating with a progressive recovery in molecular weight. These findings suggest that the reactive regenerant effectively restores the molecular weight of the binder. Mechanistically, as illustrated in Figure 7, this restoration is achieved through the ‘bridging’ effect of the regenerant: its abundant terminal epoxy groups react with the active ends of the cleaved SBS chains, thereby reconstructing the polymer’s spatial network.

### 3.2. Basic Properties

**Figure 9:**
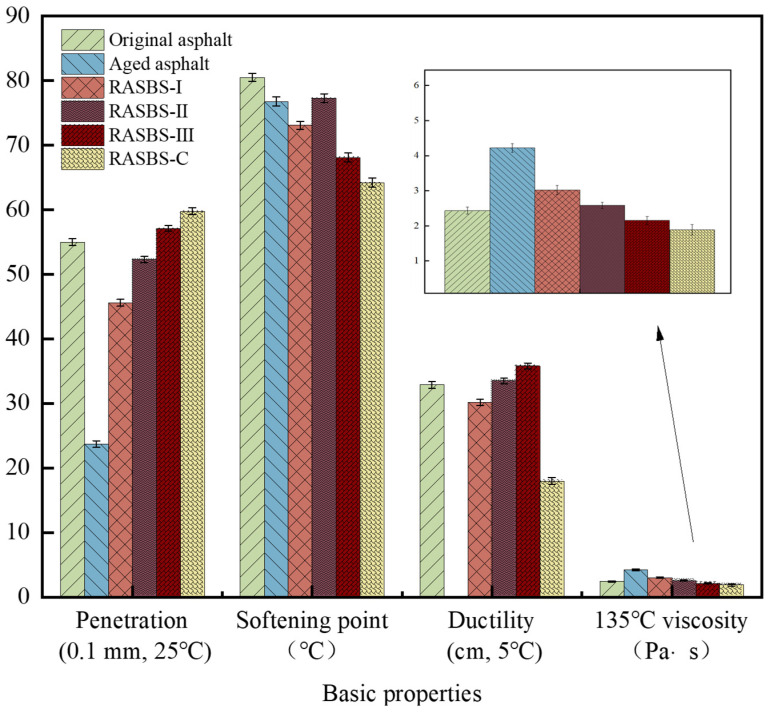
Physical properties for asphalt samples.

The conventional performance of asphalt samples are illustrated in Figure 9.

Experimental findings demonstrate that aging significantly compromises the rheological performance of SBS-modified asphalt, manifested by hardening and embrittlement. Specifically, the ductility plummeted from 32.9 cm to 0 cm, coupled with a marked rise in viscosity. Microscopic analysis attributes this degradation to the breakdown of the SBS polymer network and a disruption in the SARA colloidal balance—namely, a depletion of aromatics and an accumulation of resins and asphaltenes.

In terms of regeneration, the ZS-2 (RASBS-C) agent functions primarily as a physical softener; while it improves ductility, it excessively lowers the viscosity (to 1.89 Pa·s), failing to re-establish the structural integrity of the binder. Conversely, the reactive ZS-1 regenerant (RASBS-II at 8% dosage) successfully revitalizes the binder, achieving physical properties comparable to the virgin SBS-modified asphalt by simultaneously adjusting chemical components and reconstructing the spatial network.

Furthermore, dosage optimization for ZS-1 is critical. While increasing ZS-1 content generally enhances flexibility (penetration and ductility), an excessive dosage (10%, RASBS-III) proves detrimental. GPC analysis reveals that overdosing leads to a reduction in molecular weight and compromises colloidal stability, resulting in poor high-temperature performance and viscosity metrics. Consequently, 8% is identified as the optimal dosage, effectively balancing the restoration of the SBS network with the modulation of asphalt components.

### 3.3. High Temperature Rheological Properties

#### 3.3.1. Complex Modulus and Phase Angle

**Figure 10:**
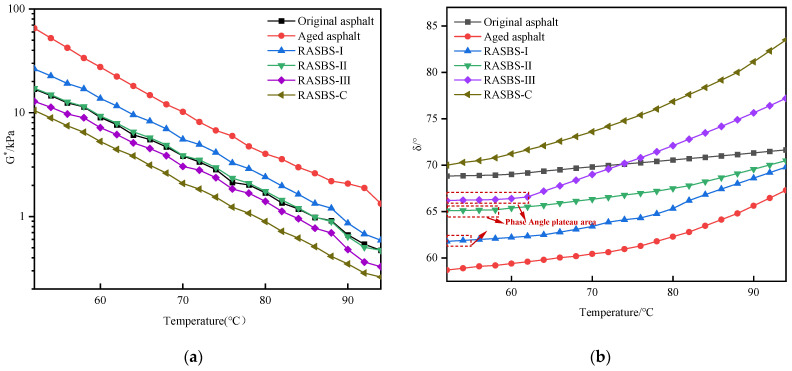
Complex modulus and phase angle of asphalt samples at 52–94 °C: (**a**) Complex modulus G*; (**b**) Phase angle *δ*.

When temperatures ranges from 52 to 94 °C, the complex modulus G* and phase angle *δ* of SBS-modified asphalt with different phases asphalt vary with temperature, as shown in Figure 10. The phase angle plateau area is an area where there is no significant change in phase angle with temperature variation. Its occurrence is mainly attributed to the elasticity contributed by the SBS phase system. However, due to the fact that the modifier content in SBS-modified asphalt is 4.5%, and it is in the dispersed phase, as the temperature increases, the elasticity of the dispersed phase is insufficient to support the elastic behavior of overall system. Therefore, in the high-temperature zone, the ramping rate of the phase angle is relatively high. Therefore, the size of the plateau area can reflect the recovering degree of the SBS modifier. A wider plateau area indicates a stronger inhibitory effect of the SBS modifier on the transition of SBS-modified asphalt from an elastic state to a viscous flow state.

Figure 10 indicates that after aging, G* of SBS-modified asphalt increases, while *δ* decreases, and the plateau area disappears. Combined with microscopic experimental results, it can be inferred that the SBS modifier degrades during the aging process, leading to significant damage to the cross-linking structure. As a result, SBS-modified asphalt gradually hardens. Comparing the samples with the addition of 8% ZS-1 and ZS-2, RASBS-C exhibits a decrease in G* and an increase in *δ*. This indicates that ZS-2 has an adverse effect on the high-temperature performance of the recycled asphalt. Upon observing the phase angle graph, the absence of a plateau area suggests that it does not have a restorative effect on SBS-modified asphalt. As the dosage of the reactive regenerant increases, G* gradually decreases, while *δ* gradually increases. The plateau area of the phase angle extends, and its manifestation becomes progressively more pronounced. Considering the conclusions drawn from microscopic mechanisms, the reason for this phenomenon is as follows: with the increase in the dosage of the regenerant, the content of SBS modifier repaired through reactions increases, which enhances the deformation resistance of the recycled asphalt at the high-temperature. However, the cross-linking action of the reactive regenerant on the degraded SBS modifier is limited. As the dosage of the regenerant increases, the impact of the light-components oil within the regenerant on the elastic state of the recycled asphalt becomes more significant, which results in insufficient elasticity of the dispersed phase to support the overall elastic behavior of the system. Consequently, in the high-temperature zone, there is a higher rate of *δ* change, and G* is lower than that of new asphalt. Hence, it has exerted a certain adverse impact on the high-temperature performance of the recycled asphalt. In a word, the reactive regenerant can effectively restore the viscoelastic properties of SBS-modified asphalt. However, there is a reasonable range for the dosage, as an excessive amount may lead to a deterioration in high-temperature and viscoelastic performance.

#### 3.3.2. Fail Temperature

**Figure 11:**
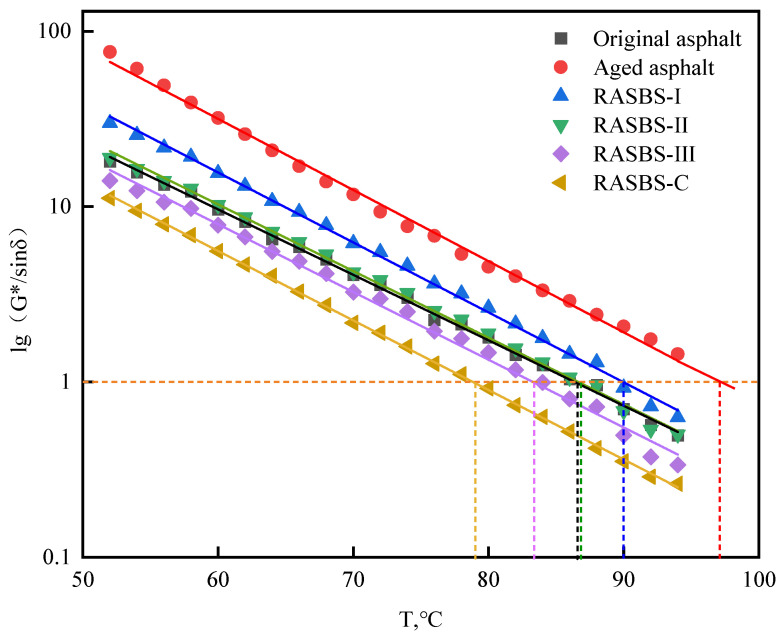
Change in rutting factor G*/sinδ with temperature of asphalt samples.

While conventional specifications rely on 60 °C metrics—such as the dynamic stability for mixtures and viscosity for binders—to gauge high-temperature performance, they fail to fully capture viscoelastic behavior under varying field temperatures and loads. To address this limitation, this study employs the failure critical temperature to evaluate resistance to permanent deformation across a temperature sweep from 52 °C to 94 °C. As depicted in Figure 11, the rutting factor drops as the temperature rises. On a semi-logarithmic scale, the rutting factor (G*/sinδ) demonstrates a significant linear dependency on temperature, validating the reliability of this evaluation method.

A linear fit was applied to the relationship between lg(G*/sinδ) and temperature (*T*) in Figure 11. The formula and parameter results obtained from this linear fit are summarized in Table 5.

In Table 5, the regression slope ‘*a*’ serves as a quantitative index for the thermal sensitivity of the binder’s viscoelasticity, where a lower absolute value implies stable performance against temperature fluctuations. The data suggests negligible disparity in thermal sensitivity across the six samples. Regarding rutting resistance, SHRP specifications mandate a minimum G*/sinδ of 1 kPa for original binders. Since this parameter correlates negatively with temperature, the specific point at which it intersects the 1 kPa threshold is defined as the failure temperature-a metric where higher values denote superior deformation resistance.

Post-aging, the failure temperature notably rises, a phenomenon driven by compositional changes (increased asphaltenes/resins and depleted aromatics) that stiffen the binder against shear. Comparative analysis shows that ZS-2 induces a sharp decline in high-temperature performance, whereas ZS-1 mitigates this loss more effectively. Supported by microscopic evidence, ZS-1’s superiority stems from its dual mechanism: rebalancing chemical fractions while simultaneously reconstructing the SBS phase structure, thereby granting RASBS-II enhanced rutting resistance. However, increasing ZS-1 dosage progressively lowers the failure temperature; this occurs because the softening effect of excess light oil eventually overrides the limited structural restoration of the degraded SBS.

### 3.4. Low Temperature Creep Characteristics

#### 3.4.1. Creep Stiffness and Rate of Change

**Figure 12:**
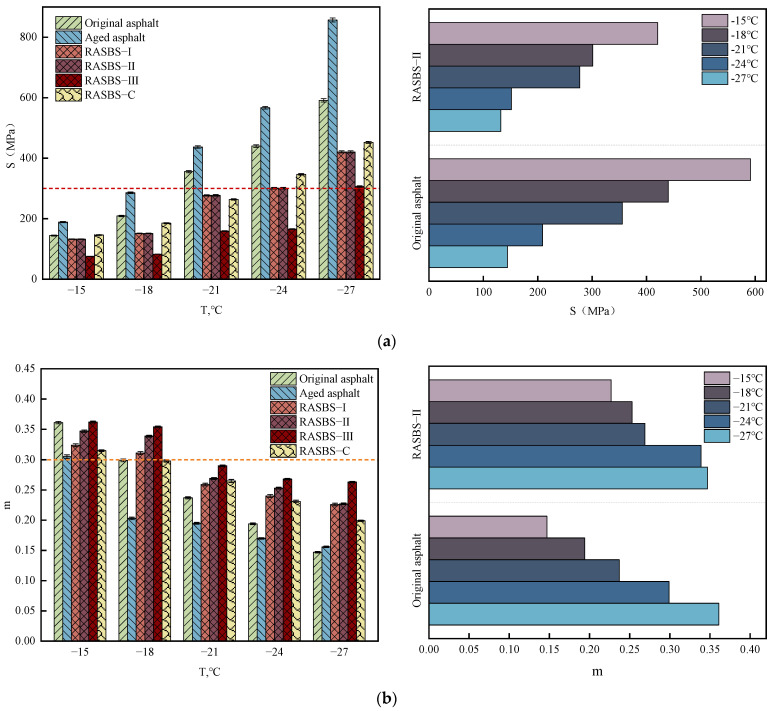
The Influence of regenerants on stiffness modulus *S* and the *m*-value of asphalt samples: (**a**) Variation in stiffness modulus *S*; (**b**) Variation in the *m*-value.

When the ambient temperature drops below 10 °C, the creep characteristics of asphalt are closely related to its low-temperature cracking resistance. Current research utilizes BBR to measure the low-temperature creep characteristics of asphalt, characterized by the creep stiffness modulus *S* and its rate of change represented by the *m*-value. The calculation formula for *S* is shown in Equation (3), where the *m*-value is the slope of *S* versus time curve at *t* = 60 s. The asphalt samples are residues after long-term aging. The low-temperature creep characteristics of the six types of asphalt are shown in Figure 12.

(3) $$S={\left(\frac{\delta }{\varepsilon }\right)}_{t,T}$$

where *S* represents the stiffness modulus of asphalt, MPa; *δ* is the stress, MPa; *ε* is the strain, %; *t* is the loading time, s; *T* is the temperature, °C.

Strategic Highway Research Program (SHRP) specifies that S of asphalt binder should not exceed 300 MPa, and the *m*-value should be not less than 0.3. As can be seen from Figure 12, after aging, S of asphalt binder samples increases sharply, and the *m*-value significantly decreases. According to the results of infrared tests, there is a certain loss of light components in aged asphalt. The low-temperature performance of SBS-modified asphalt declines after the degradation of SBS modifier. As the temperature decreases, the sample gradually hardens, increasing the risk of cracking. S and *m*-values of the original asphalt, aged asphalt, and RASBS-C only meet the specification requirements at −15 °C. In contrast, RASBS-I, RASBS-II, and RASBS-III meet the specification requirements at both −15 °C and −18 °C. Comparing samples with the addition of 8% ZS-1 and ZS-2, RASBS-II exhibits better low-temperature performance. According to the microscopic experimental results, it can be inferred that the SBS spatial network structure restored by the ZS-1 plays a ‘reinforcing’ role in the asphalt, effectively resisting cracking caused by low-temperature stress and enhancing the low-temperature cracking resistance of the recycled asphalt binder. With the increase in the dosage of ZS-1, the low-temperature performance gradually improves. The reasons for this are twofold: on one hand, the base oil effectively adjusts the colloid structure of aged asphalt; on the other hand, the epoxy-based material reconstructs the degraded SBS modifier in aged SBS-modified asphalt, which forms a stable network structure under the swelling effect of the base oil. This experiment confirms that reactive regenerant can enhance the low-temperature cracking resistance of recycled asphalt through both component adjustment and restoration of the molecular structure of SBS-modified asphalt.

#### 3.4.2. Low-Temperature Stiffness Modulus Master Curve

To elucidate rheological behaviors across a wider thermal spectrum, the construction of a master curve is a pivotal technique. Predicated on the Time-Temperature Superposition Principle (TTSP), this method involves the horizontal translation of stress relaxation modulus isotherms along the logarithmic time axis. By normalizing data to a specific reference temperature using shift factors, discrete measurements from varying temperatures and frequencies are coalesced into a continuous, smooth profile. In the context of Bending Beam Rheometer (BBR) creep testing, the time-frequency equivalency is applied. While diverse methodologies—ranging from mechanical to mathematical models—exist for master curve generation, empirical models are often favored for their computational simplicity and parameter accessibility. Consequently, this research employs the Sigmoid empirical function, defined in Equation (4), to characterize the low-temperature rheological master curve.

(4) $$\log\left|E^{*}\right|=a+\frac{b}{1+e^{-(d+g\cdot \log(f_{red}))}}$$

where *a*, *b*, *d*, and g are the regression coefficients, and *f_red_* is the reduced frequency in Hz, E* is the low-temperature stiffness modulus.

*f_red_* is obtained through Equation (5). α*_T_*(*T*) is calculated by the Williams–Landel–Ferry (WLF) equation, which is applicable to all amorphous polymers and exhibits good adaptability to asphalt materials as well, as shown in Equation (6), where *C*_1_ and *C*_2_ are model parameters, *T* is the actual test temperature, and *T*_0_ is the reference temperature.

(5) $$f_{red}=f\times \alpha_{T}(T)$$

(6) $$\log\alpha_{T}(T)=-\frac{C_{1}(T-T_{0})}{C_{2}+(T-T_{0})}$$

**Figure 13:**
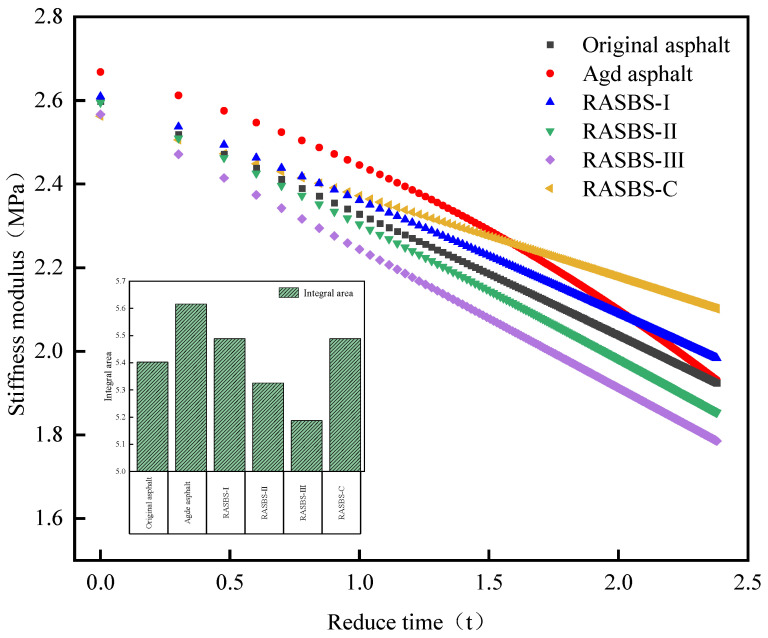
Master curves of low-temperature stiffness modulus of asphalt samples.

When establishing the master curve, −15 °C is selected as the reference temperature *T*_0_. The WLF equation is used to shift the experimental data from other temperature points to the reference temperature. The employment of the nonlinear least squares method in Excel 2024 allows obtaining both the parameters of the model and the shift factors for various temperatures simultaneously. Subsequently, graphical representations can be created to illustrate the master curves for asphalt. The master curves for each type of asphalt are shown in Figure 13.

The stiffness modulus master curve delineates the temporal evolution of binder stiffness at a specific reference temperature. A reduction in stiffness modulus signifies enhanced deformability, which translates to superior flexibility and crack resistance. Consequently, the area integrated beneath this curve serves as a quantitative metric for benchmarking low-temperature performance. A minimized integral area implies an augmented capacity for internal energy dissipation and thermal stress relaxation, thereby indicating robust anti-cracking properties. In this study, Origin 2021 software was utilized to compute these areas, with results presented in Figure 13.

As evidenced by the data, aging leads to an expansion of the integral area, reflecting compromised low-temperature performance. When contrasting 8% dosages, the ZS-2-treated sample (RASBS-C) displays inferior recovery compared to ZS-1. This limitation arises because ZS-2 acts solely as a colloidal compatibilizer without repairing the degraded SBS polymer network. Conversely, increasing the ZS-1 dosage yields a progressive reduction in the integral area. Corroborated by microscopic analysis, ZS-1 functions dually: rebalancing asphalt components and reconstructing the SBS phase. This chemically restored SBS network acts as a reinforcing phase that, through cross-linking, modifies the system’s free volume. This mechanism effectively diminishes the creep stiffness modulus, thereby fortifying the binder against thermally induced cracking.

### 3.5. Repeated Creep and Recovery Property

**Figure 14:**
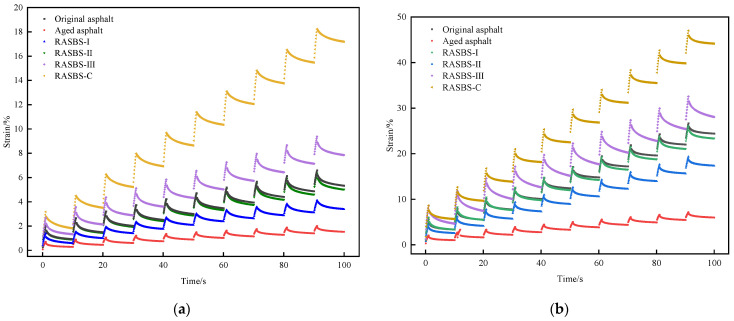
Repeated creep and recovery property of asphalt samples at different temperatures: (**a**) 60 °C; (**b**) 70 °C.

The application of MSCR tests with recovery rate (*R* at 0.1 kPa and 3.2 kPa) and non-recoverable creep compliance (*J_nr_* at 0.1 kPa and 3.2 kPa) at 60 °C and 70 °C can effectively simulate the strain patterns of the pavement under actual load conditions, reflecting the viscoelastic properties of the binder. The curves in Figure 14a,b represent the repeated creep and recovery responses of six asphalt samples under two temperatures (60 °C and 70 °C) and a stress level of 3.2 kPa for 10 cycles.

Taking the 70 °C test data as a case study, oxidative aging induced a sharp decline in total strain (from 24.42% to 5.98%), confirming the hardening effect that enhances deformation resistance. Post-regeneration, distinct behaviors were observed: the RASBS-C sample exhibited an excessive strain increase to 44.16%, whereas RASBS-I and RASBS-II showed moderate strains of 17.33% and 23.39%, respectively, closely approaching the virgin binder’s performance. Synthesizing these results with microscopic evidence, it is evident that ZS-2 functions merely as a compositional softener without repairing the SBS network, leading to the compromised high-temperature stability seen in RASBS-C.

Conversely, the reactive regenerant successfully reconstructs the SBS molecular architecture, thereby mitigating the stiffness caused by aging. However, dosage control is critical; the strain for RASBS-III rose to 28.05%, surpassing the original level. This suggests that with excessive regenerant, the plasticizing effect of the light oil fractions overrides the structural reinforcement provided by the cross-linked SBS, ultimately deteriorating the recycled binder’s resistance to permanent deformation.

**Figure 15:**
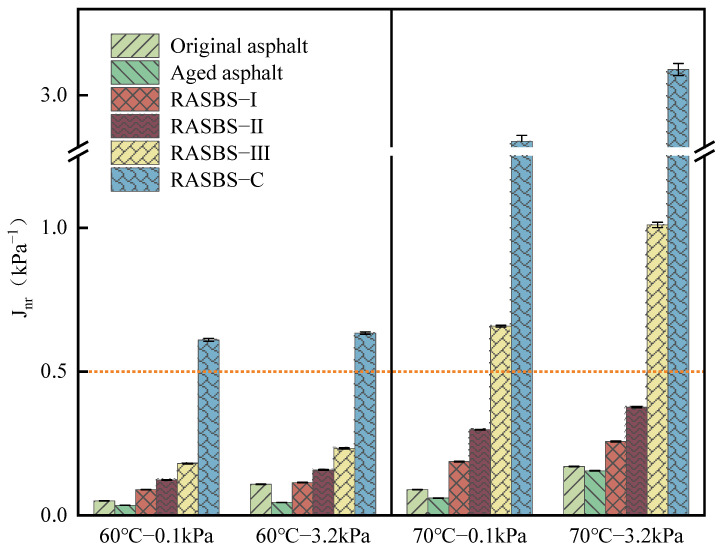
*J_nr_* of asphalt samples at 0.1kPa and 3.2kPa.

**Figure 16:**
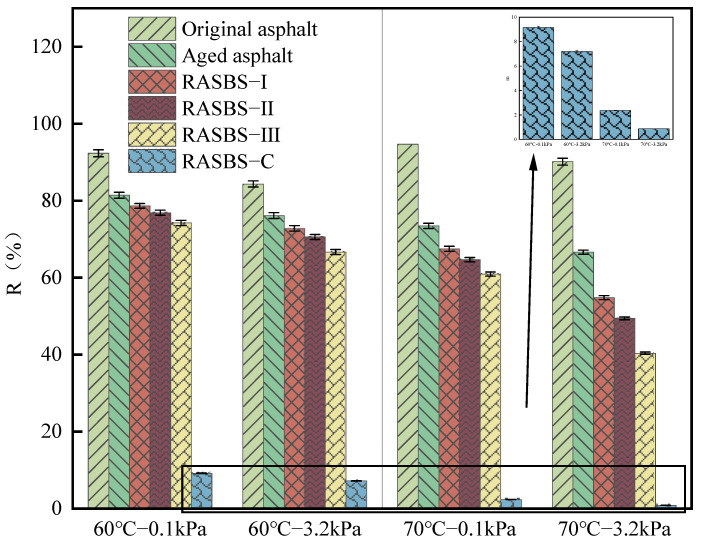
*R* of asphalt samples at 0.1 kPa and 3.2 kPa.

As illustrated in Figure 15 and Figure 16, it can be observed that after aging, both *J_nr_* and *R* values of SBS-modified asphalt decrease, indicating an enhancement in the high-temperature deformation resistance of the asphalt. However, its recovery ability under external force weakens. The addition of ZS-2 leads to a significant increase in *J_nr_* and a sharp attenuation of *R*. Considering the microscopic experimental results, it can be inferred that ZS-2 only adjusts the components but does not restore the SBS modifier that acts as reinforcement in the binder. This has a detrimental impact on the permanent deformation resistance of recycled asphalt, as it fails to restore the aged SBS-modified asphalt to its unaged state. With the increase in ZS-1 content, the *J_nr_* value increases, and the R3.2 value decreases. For RASBS-I and RASBS-II, *J_nr_*_3.2_ value increased by 46.3%, and R3.2 value decreased by 9.8%. For RASBS-II and RASBS-III, *J_nr_*_3.2_ value increased by 168.6%, and R3.2 value decreased by 18.3%. The high-temperature resistance to permanent deformation of recycled asphalt decreases as the rate of decline increases. When ZS-1 is added to aged asphalt, with the increase in ZS-1 content, *J_nr_* value increases, and R3.2 value decreases. Compared *J_nr_* and *R* of RASBS-I and RASBS-II, *J_nr_* increased by 46.3%, and *R* value decreased by 9.8%. Compared *J_nr_* and *R* of RASBS-II and RASBS-III, *J_nr_* increased by 168.6%, and *R* value decreased by 18.3%. The rate of decay of high-temperature resistance to permanent deformation of recycled asphalt increases. The reason for this is that when the reactive regenerant is added at a certain dosage, its main role in enhancing the high-temperature performance of asphalt binders is the reshaping of the SBS spatial network structure. However, when the regenerant is excessive, the dissolution of macromolecules such as asphaltene by light oil becomes the main factor, leading to a rapid decline in the resistance to permanent deformation of recycled asphalt.

Nowadays, with heavy traffic volumes, the limited time for open traffic after road maintenance places higher demands on asphalt mixtures. In this paper, *J_nr_*_3.2_ (*J_nr_* at 3.2 kPa) at 70 °C was employed to evaluate the high-temperature deformation resistance of asphalt samples. According to the high temperature performance classification requirements of AASHTO MP 19-10, the recommended range of *J_nr_*_3.2_ for asphalt binders under extreme traffic conditions at 70 °C should be no less than 0.5 kPa^−1^. The research results indicate that at 3.2 kPa and 70 °C, the *J_nr_*_3.2_ values for the samples of original asphalt, aged asphalt, RASBS-I, and RASBS-II meet the recommended values for extreme traffic. However, the *J_nr_*_3.2_ value for RASBS-III at 70 °C does not meet the recommended values for extreme traffic. Therefore, in this paper, the maximum regenerant dosage that meets the requirements for resistance to permanent deformation is determined to be 8%.

### 3.6. Analysis of Master Curve Based on 2S2P1D Model

The approach to constructing the master curve for dynamic shear rheometer (DSR) experiments is similar to that of the low-temperature master curve. It includes empirical models, mathematical models, and mechanical models. For DSR tests, the comprehensive and readily available data make it easier to collect experimental parameters. The experimental data and the quantification of relative and absolute errors for rheological models indicate that within all reduced frequency ranges, the 2S2P1D viscoelastic model demonstrates the highest accuracy in studying the rheological characteristics of asphalt materials. Compared to the Sigmoid empirical model, it provides superior adjustments to the ‘extremum’ of the master curve, allowing for a better representation and prediction of the rheological behavior of asphalt mixtures. Therefore, this paper adopts the 2S2P1D mechanical model for fitting the medium-high temperature master curve. The expression is given by Equation (7).

(7) $$E^{*}=E_{\infty }+\frac{E_{0}-E_{\infty }}{1+\mu (i\omega \tau {)}^{-k}+(i\omega \tau {)}^{-h}+(i\omega \beta \tau {)}^{-1}}$$

where *h* and *k* are dimensionless constants for the two parabolic creep elements, 0 < *k* < *h* < 1; *μ* is the dimensionless shape factor; *i* represents the imaginary unit, *i*^2^ = −1; *E*_0_ is the asymptotic glassy modulus (when *ω* → ∞), *E*_∞_ is the asymptotic static modulus (when *ω* → 0) (*E*_∞_ = 0 for asphalt binders); *β* is a dimensionless constant related to the viscosity of the sticky element, defined by Equation (8).

(8) $$\eta =(E_{g}-E_{0})\beta \tau$$

where *η* is the Newtonian viscosity; *τ* is the characteristic time. This equation is temperature-dependent and can be approximated within the temperature range of laboratory testing using methods like the WLF equation and similar shift factor approaches.

(9) $$\tau =\alpha_{T}\left(T\right)\times \tau_{0}$$

After obtaining the results of the complex modulus and phase angle, the WLF equation is applied for time-temperature superposition. The model parameters are then obtained using the least squares method in Excel, establishing well-defined master curves for the complex modulus and phase angle with a reference temperature of 20 °C. The reduced frequency can be obtained by calculating the time-temperature shift factor, and the shift factor is calculated using Equations (10) and (11).

(10) $$\omega_{r}=\alpha_{T}\left(T\right)\times \omega$$

(11) $$\log\alpha_{T}(T)=-\frac{C_{1}(T-T_{0})}{C_{2}+(T-T_{0})}$$

where *C*_1_ and *C*_2_ are model parameters, obtained through least squares fitting in Excel. *T* is the actual test temperature, *T*_0_ is the reference temperature, ω is the actual test frequency, and *ω_r_* is the reduced frequency.

**Figure 17:**
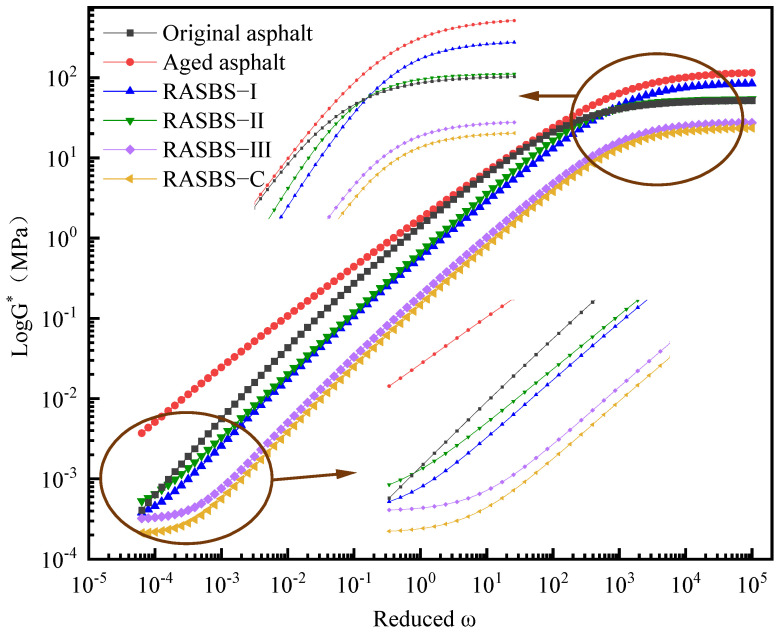
Complex modulus master curves for asphalt samples.

As illustrated in Figure 17, the aged binder exhibits an elevated modulus in the low-frequency (high-temperature) domain, signifying enhanced resistance to rutting under slow-traffic loads. In contrast, the RASBS-C sample (containing 8% ZS-2) displays a markedly lower modulus, reflecting compromised high-temperature stability. This deficit is attributed to ZS-2’s inability to reconstruct the SBS phase; it functions merely by supplementing light components, and it acts as a diluent. Conversely, the reactive regenerant (ZS-1) demonstrates a dosage-dependent nonlinearity. The modulus initially rises and then falls with increasing dosage. Specifically, RASBS-I proves insufficient for effective SBS restoration (lower modulus than RASBS-II), whereas the excessive base oil in RASBS-III induces a softening effect that overrides the structural reinforcement of the repaired SBS network, leading to performance deterioration. Thus, an optimal dosage window exists.

In the high-frequency (low-temperature) regime, aging increases stiffness, making the binder prone to cracking. However, introducing regenerants progressively lowers the complex modulus in this region. Notably, at the optimal 8% dosage (RASBS-II), the curve aligns closely with the virgin binder, confirming that the chemically reconstructed SBS network effectively restores low-temperature flexibility. While RASBS-C also shows good low-temperature compliance due to the solubilizing effect of aromatics on asphaltenes, its sacrifice of high-temperature performance renders it unsuitable for heavy-duty pavements. These rheological findings align perfectly with the proposed reaction mechanism.

### 3.7. Permeability Evaluation

**Figure 18:**
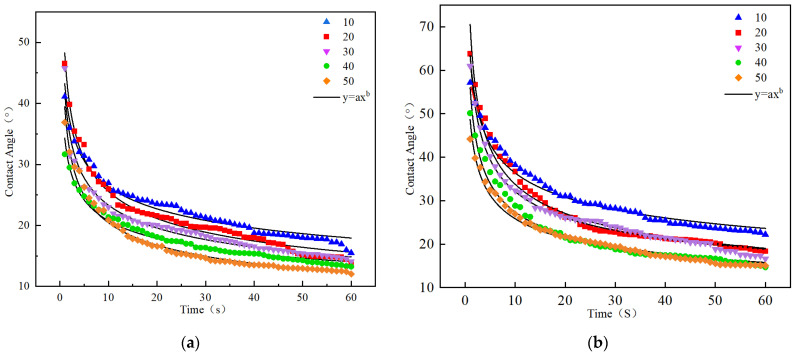
Variation in contact angle with test time at different positions in the sample tube: (**a**) Fitting curves of contact angle versus time for ZS-1; (**b**) Fitting curves of contact angle versus time for ZS-2.

Currently, most existing studies on reactive regenerants focus on the performance recovery of aged asphalt after complete integration with the regenerant, but lack an effective method to evaluate their ability to efficiently penetrate the aged asphalt layer on the surface of RAP. Based on the data obtained from the contact angle test, a scatter plot of contact angle versus time was generated and fitted, with the results presented in Figure 18.

As depicted in Figure 18, at a mixing temperature of 140 °C, the temporal evolution of the contact angle adheres to a power-law decay model (*y* = *a*·*x^b^*), achieving high correlation coefficients (*R*^2^ > 0.97) across all datasets. In this equation, the coefficients *a* and *b* govern the dynamic wetting behavior. Specifically, the coefficient *a* quantifies the instantaneous contact angle at *t* = 1 s, reflecting the initial wetting state upon asphalt-aggregate contact. The exponent *b*, on the other hand, serves as a kinetic index representing the spreading velocity.

To characterize the permeability gradient, power-law regressions were applied to contact angle data derived from the 10th through 50th sequential droplets. A higher absolute magnitude of *b* signifies a more rapid spreading rate, thereby indicating enhanced penetrability of the warm-mix regenerant. Notably, droplets positioned closer to the regenerant interface exhibit smaller initial contact angles and accelerated spreading kinetics. Consequently, the sequential droplet number acts as a proxy for depth within the sample tube, with the variation in parameter *b* mapping the extent of regenerant permeation across different layers. The detailed fitting parameters are tabulated in Table 6.

As shown in Table 6, the initial contact angles of aged asphalt at all layers with the addition of the high-permeability warm-mix regenerant are smaller than those obtained with the common regenerant. This is because the high-permeability warm-mix regenerant possesses a warm-mixing effect, enabling it to spread and wet the surface of aged asphalt more effectively, thereby reducing the surface energy of the aged asphalt and lowering the contact angle. Based on the spreading rate parameter *b*, the changes in the spreading rate of aged asphalt samples mixed with the high-permeability warm-mix regenerant are not significant in Layers 1 and 2, indicating that the regenerant has penetrated Layer 2 of the aged asphalt. In contrast, for the ordinary regenerant, the changes in spreading rate become insignificant in Layers 2 and 3, indicating that its penetration is limited to Layer 3.

**Figure 19:**
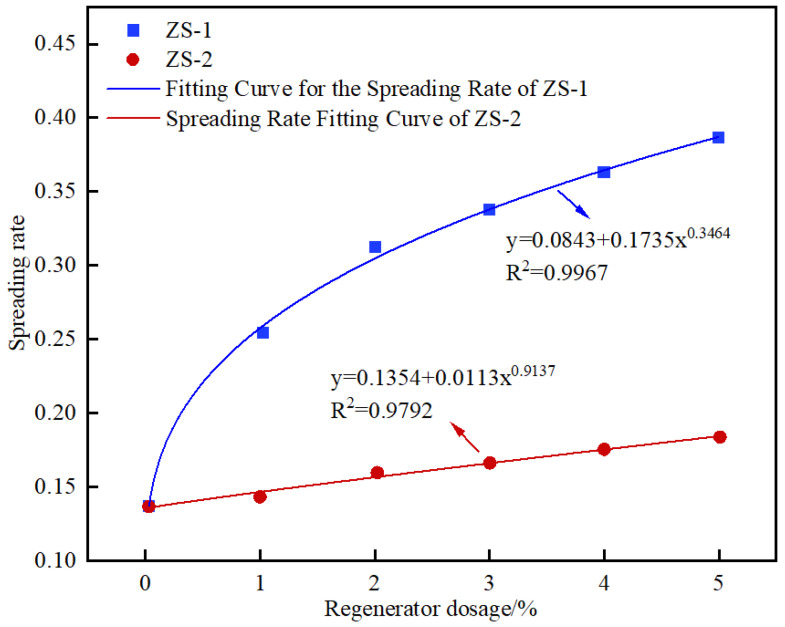
Calibration of Regenerant Penetration.

Asphalt samples were prepared by fully blending original SBS-modified asphalt with aged asphalt from the RAP surface, incorporating 0%, 1%, 2%, 3%, 4%, and 5% regenerant by weight, respectively. Scatter plots of contact angle versus time were obtained through measurement, and power function fitting was conducted to determine the spreading rate parameter *b*, with results presented in Figure 19. Subsequently, based on the spreading rate parameter *b* obtained from contact angle fitting for each layer in the corresponding sample tube, the amount of regenerant permeated in that layer was determined through interpolation. Finally, the regenerant penetration of pure aged asphalt is defined as 0%, while that of the fully miscible regenerated asphalt sample is defined as 5%. The permeability of each layer is expressed as the ratio of the penetrated regenerant dosage to the maximum regenerant penetration (i.e., that of the fully miscible sample), as given in Equation (12).

(12) $$A=\frac{B_{n}}{B_{y}}$$

where *A* denotes the regenerant permeability, %; *B_n_* represents the regenerant penetration in each layer, %; *B_y_* denotes the regenerant penetration of the fully miscible sample, which is set at 5%.

**Figure 20:**
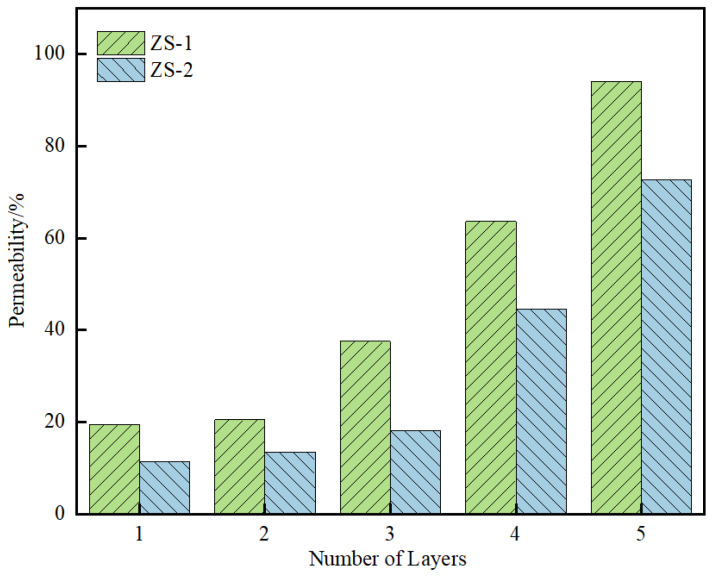
Permeability test results.

The regenerant permeability results are presented in Table 7 and illustrated in Figure 20. As shown in Figure 20, the permeability of the high-permeability warm-mix regenerant in aged asphalt is generally higher than that of the conventional regenerant, with values of 11.44% and 19.45% observed for the first layer, respectively. This indicates that the high-permeability warm-mix regenerant can more effectively restore aged asphalt on the RAP surface. Thus, the contact angle test method for quantitatively evaluating the penetration performance of this regenerant is validated and consistent with experimental observations.

## 4. Conclusions

In this paper, to investigate the influence of the reactive regenerant on the rheological response characteristics of the microscopic phase structure of aged polymer-modified asphalt, a comparison was made between two types of regenerants and different dosages of reactive regenerants in recycled asphalt samples. Firstly, the reaction mechanisms of different types of regenerants were revealed through FM, FTIR, and GPC tests. Secondly, the conventional performance, viscoelastic behavior, and creep characteristics were investigated through routine performance tests, DSR tests, and BBR tests. Finally, the contact angle test was employed to simulate the diffusion of the regenerant into aged asphalt and to evaluate its penetration performance. The following conclusions can be drawn from the above experiments:

(1) The analysis of FTIR, GPC, and FM indicates that the aging effect leads to an increase in asphalt molecular weight, but the degradation of the SBS modifier results in a decrease in the molecular weight of the SBS modifier segments. The epoxy groups in the reactive regenerant react with the active terminal groups in the degraded SBS segments, forming ester and ether bonds. This process acts as a new bridge to restructure the molecular weight distribution and spatial network structure of the degraded SBS modifier, restoring the molecular structure of the aged SBS-modified asphalt.
(2) The physical performance indicators indicate that compared to ZS-2, ZS-1 can evenly enhance various physical properties of aged SBS-modified asphalt. Moreover, with the increase in the dosage of ZS-1, the regeneration effect becomes more pronounced. Due to the higher dosage of the regenerant, the weakening effect of the light-component oil on the penetration, softening point, and viscosity of the asphalt samples becomes more pronounced. Therefore, among the three dosage levels evaluated in this study, the 8% concentration of the recycled agent demonstrated relatively superior performance.
(3) Rheological experiments indicate that ZS-1 can effectively restore the high-temperature, low-temperature rheological properties and creep recovery ability of aged SBS-modified asphalt. The reactive regenerant can reconstruct the spatial network structure of SBS-modified asphalt, acting as reinforcement in the asphalt, thereby enhancing the low-temperature cracking resistance and high-temperature deformation resistance of the recycled asphalt binder.
(4) The permeability evaluation results indicated that, compared to the ZS-2 regenerant, the ZS-1 regenerant contained a permeability-promoting modifier, enabling superior diffusion performance in aged SBS-modified asphalt. Specifically, the permeability rates of the first layer were 11.44% and 19.45% for ZS-2 and ZS-1, respectively. Therefore, the reactive regenerant with enhanced permeability should be selected as the regenerating agent for aged SBS-modified asphalt to promote the reaction between epoxy groups and active end groups resulting from aging-induced bond scission.
(5) Compared with conventional component-mixed regenerants, the SBS cross-linked network self-healing regenerant effectively restores the original SBS polymer network architecture and comprehensively recovers the rheological, mechanical, and aging resistance properties of reclaimed asphalt—without compromising its high-temperature stability. This advancement facilitates the incorporation of SBS-modified reclaimed asphalt pavement (RAP) into surface and intermediate pavement layers, thereby enabling high-value, performance-driven utilization of asphalt-based solid waste.

## Figures and Tables

**Table 1:** Properties of base asphalt.

Items		Result	Specification Value
Penetration/(0.1 mm, 25 °C)		72	60–80
Ductility/(cm, 5 °C)		47.6	≥100
Softening point/°C		126	≥46
Flash point/°C		324	≥260
Solubility/%		99.8	≥99.5
TFOT (163 °C, 5 h)	Mass loss/%	0.163	±0.8
	Penetration ratio/%	64.1	≥61
	Ductility/(cm, 15 °C)	38.5	≥15

**Table 2:** Properties of SBS-modified asphalt.

Items		Result	Specification Value
Penetration/(0.1 mm, 25 °C)		55	40–70
Ductility/(cm, 5 °C)		32.9	≥25
Softening point/°C		80.5	≥70
60 °C dynamic viscosity/Pa⋅s		33,265	≥20,000
135 °C viscosity/Pa⋅s		2.43	≤3
Elastic recovery/%		98.1	≥80
Segregation/°C		0.8	≤2.5
TFOT (163 °C, 5 h)	Mass loss/%	0.04	±0.5
	Penetration ratio/%	85.7	≥65
	Ductility/(cm, 5 °C)	24.9	≥15
Solubility/%		99.4	≥99
Flash point/°C		315	≥245

**Table 3:** Performance indicators of SBS-modified asphalt after RTFOT and PAV.

Items	Result	Test Methods (ASTM)
Penetration/(0.1 mm, 25 °C)	23.7	D5, 2013
Ductility/(cm, 5 °C)	-	D113, 2017
Softening point/°C	76.8	D36, 2009
135 °C viscosity/Pa⋅s	4.22	D4402, 2023

**Table 4:** Properties of the regenerants.

Items		Result		Specification Value
		ZS-1	ZS-2	
60 °C viscosity/(cSt, 60 °C)		135	172	50–175
Saturates content/%		19	15	≤30
Aromatic content/%		46	37	Measured
TFOT (163 °C, 5 h)	Mass variation/%	−1.99	−1.65	±4
	Aromatic content	1.4	1.6	<3
Flash point/°C		235	238	>220

**Table 5:** Linear regression equation of lg(G*/sinδ) and temperature for asphalt samples.

Types of Asphalt	$\mathrm{lg}(\frac{G*}{\sin\delta })=aT+b$	Parameter *a*	Parameter *b*	Correlation Index R2	Failure Temperature/°C
Original asphalt	$\mathrm{lg}(\frac{G*}{\sin\delta })=-0.0373T+3.2246$	0.0373	3.2246	0.9982	86.45
Aged asphalt	$\mathrm{lg}(\frac{G*}{\sin\delta })=-0.0406T+3.9352$	0.0406	3.9352	0.9963	96.93
RASBS-I	$\mathrm{lg}(\frac{G*}{\sin\delta })=-0.0399T+3.5884$	0.0399	3.5884	0.9975	89.93
RASBS-II	$\mathrm{lg}(\frac{G*}{\sin\delta })=-0.0380T+3.2932$	0.0380	3.2932	0.9972	86.66
RASBS-III	$\mathrm{lg}(\frac{G*}{\sin\delta })=-0.0386T+3.2169$	0.0386	3.2169	0.9939	83.34
RASBS-C	$\mathrm{lg}(\frac{G*}{\sin\delta })=-0.0396T+3.1253$	0.0396	3.1253	0.9995	78.92

**Table 6:** Fitting equations and Parameters of Contact Angle vs. Time at Different Positions in Sample Tubes.

Temperature /°C	Types of Regenerant	Sample Position /Droplet	*y* = *a·x^b^*		
			*a*	*b*	*R* ^2^
140	High-permeability warm-mix regenerant	10	95.923	−0.251	0.987
		20	92.171	−0.255	0.991
		30	90.420	−0.302	0.984
		40	80.974	−0.345	0.988
		50	71.549	−0.378	0.992
	Conventional regenerant	10	104.021	−0.137	0.999
		20	104.712	−0.139	0.998
		30	106.024	−0.143	0.995
		40	111.374	−0.159	0.998
		50	128.752	−0.171	0.998

**Table 7:** Test Results of Regenerant Permeability for Different Types of Regenerants.

Test Samples		Penetrated Regenerant Dosage /%		Permeability /%	
		ZS-1	ZS-2	ZS-1	ZS-2
Aged SBS-modified asphalt	Aged asphalt	0	0	0	0
	Layer 1	0.97	0.57	19.45	11.44
	Layer 2	1.03	0.68	20.51	13.50
	Layer 3	1.88	0.91	37.54	18.18
	Layer 4	3.18	2.23	63.65	44.58
	Layer 5	4.70	3.64	94.03	72.76

## Data Availability

The original contributions presented in this study are included in the article. Further inquiries can be directed to the corresponding author.
